# Exploring care-seeking practices within a family mid-upper arm circumference approach in South Sudan: a mixed-methods prospective study

**DOI:** 10.1186/s12889-025-23010-w

**Published:** 2025-05-13

**Authors:** Sarah Bauler, Chiara Altare, Sule Ismail, Daniel Atem, Sandra Banks, Prachi Srivastava, Julia Hussian, Emily Lyles, Eva Leidman, Shannon Doocy

**Affiliations:** 1 World Vision International, London, UK; 2 Johns Hopkins Bloomberg School of Public Health, Baltimore, MD USA; 3 Integral Global Consulting, Tucker, GA USA; 4 US Centers for Disease Control and Prevention, Atlanta, GA USA; 5 World Vision South Sudan, Juba, South Sudan

**Keywords:** Child wasting, Family mid-upper arm circumference (MUAC) approach, Care seeking, Acute malnutrition, Low- and middle-income countries (LMICs), South Sudan, Africa

## Abstract

**Background:**

Despite the growing adoption of the Family Mid-Upper Arm Circumference (MUAC) approach to empower caregivers in detecting child malnutrition, limited evidence exists on whether caregivers act on identified cases by seeking care and factors influencing their decisions. Most research has focused on the accuracy of caregiver MUAC measurements, leaving a gap in understanding behavioral, social, emotional, and contextual barriers to care-seeking. Addressing this gap is critical for informing interventions to ensure early detection translates into timely treatment. This study aimed to explore the barriers and facilitators influencing care-seeking practices within a Family MUAC program in South Sudan.

**Methods:**

We conducted a mixed-methods, prospective, non-randomized study in Central Equatoria and Warrap States, South Sudan, between March 2022 and January 2023. We enrolled 2,893 children aged 5–53 months and trained their caregivers on using MUAC tapes. Caregivers were followed for 8 months, including three monitoring visits and baseline/endline surveys, capturing self-reported care-seeking practices. Qualitative data were obtained through 20 focus group discussions (FGDs) with caregivers, using the Health Belief Model as a theoretical framework to explore perceptions, barriers, and enablers of care-seeking. A combined deductive and inductive coding approach was used for thematic analysis.

**Results:**

Among children identified with wasting using MUAC tapes, 86.5% of caregivers sought care, with significantly higher rates in Warrap (97.6%) than Central Equatoria (79.4%) (*p* < 0.008). Barriers to care-seeking included distance to health facilities (18.9%), transportation costs (11.3%), and treatment costs (9.4%). Qualitative findings revealed additional challenges such as social stigma, lack of knowledge about where to seek care, and negative experiences with health workers. Despite some caregivers reporting a lack of encouragement, most valued the MUAC tapes, used them weekly, and were confident in their ability to take accurate measurements.

**Conclusions:**

Policies and programmatic interventions should consider integrating Family MUAC programs with community-based financial initiatives like savings groups to address financial barriers. Tailoring interventions to rural and urban contexts through formative research can enhance program effectiveness, while training health workers in compassionate care may improve caregiver trust and increase care-seeking rates. Strengthening these areas can maximize the impact of Family MUAC and improve child health outcomes.

**Trial registration:**

N/A.

**Supplementary Information:**

The online version contains supplementary material available at 10.1186/s12889-025-23010-w.

## Background

Children under five years of age are particularly vulnerable to wasting, a form of acute malnutrition characterized by severe weight loss, weakened immune system, and increased risk for developmental delays, illness, and death [1]. Yet, despite the fact that over one-third of child deaths are attributable to wasting [2], 80% of more than 50 million children with moderate and severe wasting globally do not receive care [3]. A significant barrier to accessing care is poor caregiver healthcare-seeking practices, which significantly contribute to child morbidity and mortality in sub-Saharan Africa [4]. While the evidence about caregiver healthcare-seeking behavior in South Sudan is limited, studies from Ethiopia and Kenya identified low perceived severity of an illness [5] and distance from a health facility as barriers that impact the likelihood of caregivers seeking care for the sick child [6]. Where caregivers do not perceive the severity of wasting, financial and logistic barriers such as distance may delay care seeking. Survival outcomes are poorer when care is delayed and children present with more severe forms of wasting [7].

One strategy for early identification of wasted children at the community level is the Family Mid-Upper Arm Circumference (MUAC) approach. The Family MUAC approach empowers caregivers by training them to screen their children for wasting by using a simplified, color-coded MUAC tape and testing for edema [8, 9]. In low- and middle-income countries, the Family MUAC approach was introduced in 2012 for early detection of child wasting cases at the community level to increase the number of children referred for treatment at a community management of acute malnutrition (CMAM) program [10, 11].

The rationale for this study is to address a critical gap in existing research by exploring the care-seeking behaviors of caregivers using the Family MUAC approach, rather than focusing solely on the accuracy of measurements or the performance of different MUAC tapes. While previous studies have established that caregivers can effectively measure their children’s nutritional status using MUAC tapes [12–14], there is limited understanding of the factors that influence whether caregivers act on these measurements by seeking appropriate medical care. This study aims to fill that gap by exploring barriers and facilitators to care seeking, including social, financial, and contextual factors, to ensure that the benefits of the Family MUAC approach translate into improved health and nutrition outcomes for children​. To achieve this, we conducted household surveys at baseline and endlin, including three monitoring visits, complemented by focus group discussions (FGDs) with caregivers participating in the Family MUAC program. This mixed-methods approach provided a comprehensive understanding of the factors that support or hinder healthcare-seeking behaviors in both urban and rural communities in South Sudan.

## Methods

### Study design and setting

We employed an explanatory sequential, mixed-methods approach to bridge the research gap by combining quantitative and qualitative data to gain a comprehensive understanding of care-seeking practices. This approach offers significant advantages, as it enables the measurement of trends and statistical relationships through quantitative data, while qualitative data provides deeper insights into contextual, social, and emotional factors influencing care-seeking behaviors. The qualitative component of this study is part of a larger mixed-methods, three-group, prospective non-randomized study that examined caregivers’ use of three different MUAC tape types—UNICEF 2009, UNICEF 2020, and Goal MAMI tapes—in South Sudan [15]. In this paper, we present a subset of the study’s data, focusing specifically on the barriers and facilitators to MUAC tape utilization. The qualitative findings enriched and complemented the quantitative data by offering context to the identified barriers and enablers of care-seeking behaviors, while also uncovering social, emotional, and contextual dimensions that were not captured in household surveys and monitoring visits. This integrated approach strengthened the validity and reliability of our findings through triangulation, provided richer insights by incorporating diverse perspectives, and facilitated a more holistic and nuanced analysis to inform context-specific Family MUAC interventions. The study was conducted in Central Equatoria (Juba County) and Warrap (Twic County) states, covering seven CMAM program sites operated by World Vision. Warrap represents a predominantly rural area prone to flooding, whereas Central Equatoria is more urbanized with better access to healthcare and transportation services.

### Data collection

The total sample size for the study was 2,100 households (700 per group) and was identified as sufficient to detect differences ≥ 10% in the false negative rate. Inclusion criteria included residence within selected communities in the CMAM catchment area and the presence of a 5-to-53 month-old child in the household. Children who were acutely malnourished at enrolment were excluded from the study and referred to the CMAM program for treatment. Caregivers were invited to attend a 1 to 2-hour training site (community centers, CMAM sites, or health facilities) by World Vision nutrition staff, provided with a MUAC tape, and trained on its use; at the end of the session, program staff confirmed that caregivers could take accurate measurements of their children.

For caregivers that consented to be enrolled in the study, quantitative data were collected at households five times over the 8-month study period: at baseline, in 3 monitoring visits (months 2, 4, 6), and at endline (month 8). The baseline survey questionnaire (Annex B) included individual characteristics (mother and child) and household characteristics (e.g., size and displacement status), while instruments for monitoring visits and endline were more limited and focused on food security indicators, tape utilization, and child anthropometry (MUAC measured by the evaluation team). For more detail describing the methods used in the quantitative component, please see our paper “Caregiver use of MUAC tapes in South Sudan: A three-group prospective comparison.” [15].

FGD participants were purposively selected based on predefined criteria to ensure a diverse representation of experiences and perspectives from all three study arms. These criteria included households with malnourished children 5–53 months, households that had sought care at CMAM sites, and those that had not sought care. Potential participants were identified from lists provided by study coordinators and were contacted to confirm their willingness to participate. The recruitment process aimed to secure seven participants for each FGD to account for potential no-shows, ensuring a final group of six participants. The training was conducted for FGD facilitators and note-takers on September 5, 2022, on the study objectives, guiding discussions using open-ended and non-leading questions, probing techniques, participant recruitment strategy, group dynamics, ensuring proper consent and data collection procedures, and minimizing the risk for COVID-19. Our sequential study design involved using the quantitative preliminary findings to inform the FGD guide, which included questions exploring caregivers’ trust in MUAC measurements, changes in care-seeking behavior, treatment center experiences, and perceptions of respect and care from Ministry of Health (MOH) staff and community nutrition volunteers (CNVs).

A total of 20 FGDs were conducted from September 7 to 30, 2022; 12 of the sessions were organized by study arm (such that individuals in a given FGD were all assigned to use the same type of MUAC tape), and eight were organized with members across study arms to allow for a comparison of MUAC tapes. The FGDs were conducted indoors at health facilities or community centers, selected based on their proximity to participating caregivers, to ensure privacy and enhance the quality of recorded discussions. Before starting discussions, the facilitator read the consent script to inform participants of their voluntary participation, right to withdraw without consequences, and right to skip uncomfortable questions, while also seeking permission to record the discussion without recording names to ensure confidentiality. Each FGD had six participants and was conducted in Juba Arabic. FGDs were recorded, with participants’ agreement, transcribed, and translated into English by the note-taker and facilitator.

### Data analysis

Descriptive statistics were used to examine baseline characteristics and cumulative care seeking among all children who completed the study [defined as having completed baseline, endline, and two or more monitoring visits] and their respective households. Care seeking was disaggregated by nutritional status. Statistical significance of differences by location was assessed using chi-square and *t*-test methods, and analysis was conducted in Stata 15 [16].

For the qualitative component, we utilized the Health Belief Model [17] as our theoretical framework and a deductive and inductive approach to coding. Descriptive, process, emotional, value, and concept codes were developed to capture barriers and enablers to desired childcare practices, perceived self-efficacy, and cultural beliefs and perspectives related to caregiver practices. Once the transcripts were coded and coded segment reports were extracted, a saturation grid was developed to map and count the occurrence of emerging themes and sub-themes. We primarily used horizontal coding to identify patterns and themes across participants’ responses, allowing for a comprehensive analysis of similarities and variations in the data. To ensure the rigor and trustworthiness of our analysis, coding consistency was verified through team discussions and iterative refinement of the codebook. Qualitative data were managed and coded in MAXQDA [18]. The qualitative research findings were triangulated with our quantitative data analysis and literature review to enhance the credibility of the findings. The study’s findings are rooted in the specific context of South Sudan, but the use of rich descriptions of participants’ childcare-seeking practices aims to facilitate transferability, enabling readers to assess the relevance of these results to their own contexts.

### Ethics approval and consent to participate

The study was reviewed and approved by the South Sudan Ministry of Health Ethics Committee and Institutional Review Board at Johns Hopkins Bloomberg School of Public Health (IRB Number 00016618). Informed consent was obtained from all caregivers to participate in the study. This activity was reviewed by the United States Centers for Disease Control and Prevention (CDC) and was conducted in a manner consistent with applicable federal law and CDC policy. All study activities involving human subjects were conducted in accordance with the ethical standards of the institutional and national research committees and with the 1964 Helsinki Declaration and its subsequent amendments.

## Results

### Quantitative results

#### Baseline description of children and households

A total of 2,893 children from 2,185 households were enrolled in the study (Table 1). Of those, 2,401 (83.0%) children from 1,826 households completed baseline, endline, and two or more monitoring visits and were included in the analysis. Study completion rates were significantly higher in Warrap (98.6%) than in Central Equatoria (67.5%), where the population is more mobile (*p* < 0.001). The median age at enrollment was 28.9 months (mean 29.5 months, IQR 17.1–39.1 months), and 51.4% of children were female. MUAC at enrollment was 14.6 cm (mean 14.5 cm, IQR 13.8–15.4 cm) on average and significantly higher in Central Equatoria (14.9 cm) than in Warrap (14.4 cm). More households in Central Equatoria (18.3%) were currently displaced compared to those in Warrap (0.7%), and female-headed households were more common in Warrap (31.8%) than in Central Equatoria (27.3%). Only 4.3% of households received food or cash assistance the month before enrollment. Severe hunger was significantly higher in Warrap (24.0%) than in Central Equatoria (5.4%), but the percentage of children ever wasted during the study period was higher in Central Equatoria (5.4%) than in Warrap (3.9%).

**Table 1:** Participant characteristics at baseline by location and study group

		Overall (*N* = 2,401)	By Location		
			Central Equatoria	Warrap	*p*-value
			(*n* = 981)	(*n* = 1,420)	
Participant [child] characteristics					
Female		1233 (51.4%)	522 (53.2%)	711 (50.1%)	0.130
Age (in months)	Mean (SD)	28.9 (13.5)	28.9 (13.4)	29.0 (13.5)	0.772
6 to < 24 months		846 (35.2%)	358 (36.5%)	488 (34.4%)	0.283
≥ 24 months		1555 (64.8%)	623 (63.5%)	932 (65.6%)	
MUAC at enrollment (cm)^1^	Mean (SD)	14.6 (1.1)	14.9 (1.1)	14.4 (1.0)	***< 0.001***
Prior acute malnutrition diagnosis		460 (19.2%)	157 (16.0%)	303 (21.3%)	**0.001**
Household characteristics		(*N* = 1,826)	(*n* = 750)	(*n* = 1,076)	
Household size	Mean (SD)	7.5 (2.5)	7.4 (2.8)	7.6 (2.2)	0.073
Household is currently displaced		144 (7.9%)	137 (18.3%)	7 (0.7%)	***< 0.001***
Female-headed household		547 (30.0%)	205 (27.3%)	342 (31.8%)	**0.041**
Received food assistance (past month)^2^		79 (4.3%)	7 (0.9%)	72 (6.7%)	***< 0.001***
Household Hunger Scale	Mean (SD)	2.8 (1.4)	2.5 (1.2)	3.1 (1.4)	***< 0.001***
Little to no hunger		248 (13.9%)	136 (18.9%)	112 (10.6%)	***< 0.001***
Moderate hunger		1240 (69.6%)	546 (75.7%)	694 (65.5%)	
Severe hunger		293 (16.5%)	39 (5.4%)	254 (24.0%)	
Caregiver characteristics					
Caregiver age (years) ^3^	Mean (SD)	28.0 (7.8)	29.1 (7.8)	27.0 (7.7)	***< 0.001***
≤ 18 years		204 (11.3%)	76 (10.5%)	128 (11.9%)	***< 0.001***
19–31 years		331 (18.4%)	136 (18.7%)	195 (18.1%)	
32–37 years		1125 (62.4%)	484 (66.6%)	641 (59.6%)	
38–48 years		43 (2.4%)	15 (2.1%)	28 (2.6%)	
49 + years		99 (5.5%)	16 (2.2%)	83 (7.7%)	
Highest Level of Education Completed					
Less than primary		1440 (82.0%)	549 (73.2%)	891 (88.6%)	***< 0.001***
Primary		181 (10.3%)	94 (12.5%)	87 (8.6%)	
Secondary or higher		135 (7.7%)	107 (14.3%)	28 (2.8%)	

^1^per study team measurement; ^2^includes in-kind food assistance and cash assistance that could be used to purchase food; ^3^ specific age (in years) only reported by 1,584 (86.7%) respondents and age categories were developed according to local memorable events to facilitate more reliable recall

#### Care-seeking behavior

Table 2 shows care-seeking behavior among study participants. Slightly more than half of the respondents (55.3%) initiated care seeking if they were worried about their child’s nutrition or being too thin. Among children identified with wasting (3.5%) by caregivers, 91.3% initiated care seeking, although care seeking was higher in Warrap (97.4%) than in Central Equatoria (86.8%) (*p* < 0.008) (Table 2). CMAM enrollment was also higher among wasted children in Warrap (71.8%) than in Central Equatoria (41.5%) (*p* < 0.002); average CMAM enrollment for both states was 54.3%. When evaluating health outreach coverage, 14.9% of caregivers with a wasted child were visited by a CNV or community health worker (CHW), and only 14.7% were screened with a MUAC tape by CHW/CNV. Similar to CMAM enrollment, a higher percentage of wasted children were screened by a CHW/CNV in Warrap (17.3%) than in Central Equatoria (11.1%) (*p* < 0.001).

**Table 2:** Care seeking among wasted children and coverage of community health outreach activities

	Overall	By Location		
		Central Equatoria	Warrap	*p*-value
Child wasting status^1^				
*N*	2401	981	1420	
Not wasted	95.5%	94.6%	96.1%	0.194
Wasted - Identified	3.5%	4.2%	3.1%	
Wasted - Not identified	1.0%	1.2%	0.8%	
Care seeking among wasted children^2^				
*N*	92	53	39	
Household initiated care seeking	91.3%	86.8%	97.4%	0.073
CMAM enrollment	54.3%	41.5%	71.8%	**0.004**
Health outreach coverage^3^				
*N*	1826	750	1076	
Visit by CNV or CHW	14.9%	12.0%	16.9%	**0.004**
CNV/CHW MUAC screening	14.7%	11.1%	17.3%	***< 0.001***

^1^child ever identified as wasted by study team (based on 4 visits over 8 months), and caregiver identification of wasted children compared to study team gold standard (MUAC < 12.5 cm defined as wasted); ^2^once or more during the study period; ^3^once or more during the study period, household level

#### Reasons for not seeking care– children ever identified as wasted

Among caregivers with children who were ever identified as wasted during the study period, the most cited reasons for not seeking care were distance (20%), transportation cost (13.3%), and treatment cost (8.9%) (Table 3). While 12.5% of caregivers in Central Equatoria reported not knowing where to seek care, no caregivers in Warrap reported this as a reason. Other reasons for not seeking care included the family did not think it was important to seek care (8.9%), the caregiver did not have the time (4.4%), and the caregiver did not like the quality of care (2.2%).

**Table 3:** Reasons for not seeking care among children ever identified as wasted by project staff

	Overall (*N* = 45)	By Location		
		Central Equatoria	Warrap	*p*-value
		(*n* = 32)	(*n* = 13)	
**Reason for not taking child for care**				
Child was not identified as malnourished by the caregiver	0 (0.0%)	0 (0.0%)	0 (0.0%)	Insufficient sample size (< 30 in each group)
Did not know where to go	4 (8.9%)	4 (12.5%)	0 (0.0%)	
Did not like the quality of care	1 (2.2%)	1 (3.1%)	0 (0.0%)	
Treatment center was far away	9 (20.0%)	7 (21.9%)	2 (15.4%)	
Could not reach treatment center (e.g., roads impassible, poor security)	0 (0.0%)	0 (0.0%)	0 (0.0%)	
Too expensive [transportation]	6 (13.3%)	6 (18.8%)	0 (0.0%)	
Too expensive [treatment cost]	4 (8.9%)	4 (12.5%)	0 (0.0%)	
Treatment center hours are not convenient	0 (0.0%)	0 (0.0%)	0 (0.0%)	
Did not have time / too busy	2 (4.4%)	2 (6.2%)	0 (0.0%)	
Family did not think it was important to go	4 (8.9%)	3 (9.4%)	1 (7.7%)	
Child “did not fall ill”	1 (2.2%)	1 (3.1%)	0 (0.0%)	
Other	2 (4.4%)	0 (0.0%)	2 (15.4%)	
**Did not seek care at least one time during follow up but no reason ever reported**	16 (35.6%)	8 (25.0%)	8 (61.5%)	
**Number of reasons ever reported for not seeking care (among children ever wasted who did not seek care at least 1x during follow-up)**				
No reason ever reported	16 (35.6%)	8 (25.0%)	8 (61.5%)	
One reason ever reported	25 (55.6%)	20 (62.5%)	5 (38.5%)	
Two reasons reported (at different follow-up times)	4 (8.9%)	4 (12.5%)	0 (0.0%)	

### Qualitative results

#### Key influencers: who encourages and discourages taking MUAC measurements

To understand caregivers’ social environments and their experiences and perceptions of taking child MUAC measurements, we investigated factors influencing the desired behavior (i.e., taking weekly MUAC tape measurements). We found nearly an equal distribution of caregivers who reported someone encouraging them to take a MUAC measurement and those who did not receive encouragement. Caregivers received positive encouragement from World Vision training program staff, the MOH CNVs, spouses, other family members (e.g., in-laws, grandparents, siblings, cousins, aunts/uncles), and, occasionally, neighbors. However, caregivers primarily derived encouragement from their own initiative, as one respondent commented,

*For me*,* no one encourages me. I encourage myself alone*.

Discouragement primarily emanated from neighbors, family members, and other individuals in close proximity. Despite discouragement, many respondents exhibited self-confidence in performing measurements and appeared unaffected by discouraging influences. One caregiver shared an experience of discouragement, stating,

*I was once discouraged*,* but I did not accept it because I want to always know the status of my child*.

#### Self-efficacy: caregiver confidence in taking MUAC measurements

Most respondents expressed confidence in their ability to conduct MUAC measurements. Nevertheless, a minority exhibited hesitancy in their capacity to accurately take and interpret the measurements. Specifically, the millimeter increments, represented by small lines, were identified as a source of confusion. One caregiver remarked,

*I don’t have full confidence [in] the small lines; I don’t measure them.*

#### Experience at a nutrition treatment center

We explored caregiver experience at nutrition treatment centers to elucidate caregivers’ perceptions while seeking care for their children at these facilities. The experiences varied considerably; however, most respondents reported that their child was admitted and/or received treatment or were aware of others who had been admitted to a center.

Financial insecurity emerged as a significant barrier to accessing care for several respondents. One caregiver remarked,

*If you don’t have anything (money)*,* the people in the clinic refuse to do anything.*

Additionally, some participants mentioned that their child was denied access to a center or had heard of other children being turned away despite concerning measurements. In one instance, a caregiver recounted,

*The other time my child was very sick*,* I went [to health facility X] because I measured the child*,* and the measurement fell [on a bad reading]. When I got there*,* those people told me the child’s weight is good*,* and we came back with the child*.

In such cases, respondents visited the hospital, but their child was not admitted as they were deemed healthy enough to return home. Furthermore, a few caregivers reported negative experiences at treatment centers or hospitals. One caregiver commented,

*The hospital people are not good sometimes. They will see the situation of the child (is bad) but will throw a word at you*.

#### Identifying when a child is malnourished

The Health Belief Model was used to examine caregivers’ ability to recognize when their child is sick or malnourished. Common alert signs included conditions such as diarrhea, vomiting, weakness, and poor appetite. One respondent stated,

*If the child is not eating*,* I will immediately know that the child is sick*.

Similarly, another participant commented,

“*If my child has diarrhea*,* then I can measure to know*,* and if he is sick*,* then I will take the child to the hospital*”.

#### Care-seeking behavior when a child is malnourished

With respect to caregivers’ actions following the identification of their child as sick or malnourished, the majority of caregivers either increased the frequency with which they measured MUAC to monitor children’s health status or sought medical assistance at hospitals or treatment centers. For instance, one caregiver described her experience utilizing the MUAC tape as a resource for increasing her involvement in children’s nutritional/health status,

*I walked around to measure children; I measure my children every week; during my measurement*,* I found three children to be weak*,* and these children were taken to Goal [referring to World Vision site]*.

Many caregivers utilized the MUAC tape in combination with increasing their child’s food consumption, especially if they noticed a decline in their child’s MUAC reading. A caregiver elaborated further by stating:

I measured my child and found him in yellow color; then I gave him food to eat, like fish and milk.

Many caregivers commented on the profound emotional impact experienced by family members and caregivers when a child is visibly unwell, specifically noting the heartfelt commitment of families to improve their child’s health status.

#### Cultural beliefs and social norms that influence care-seeking practice

The focus of investigating the cultural beliefs and social norms aimed to explore the obstacles facing families, caregivers, and communities participating in the Family MUAC pilot study. A notable barrier cited by several FGD participants was the negative stigma inflicted by community members on a child’s health status. One participant described their experience as follows:

*When you carry a malnourished child*,* people turn to disrespect you*,* but when yours is healthy (fat)*,* then people respect and like you.*

Other participants highlighted the lack of interest and enthusiasm for the MUAC program and tapes by other caregivers:

….even if they were given the tapes, they don’t measure the children. Even if the child is very weak, they will not (be) taken to the hospital. If you go to measure and talk to them, just like what my sister said, they will ask you how you know. And they don’t want to measure the children.

The community expressed appreciation toward the MUAC tape facilitators and training staff for increasing their access to education, specifically by providing them with skills they can use to feel further engaged with their child’s health. One respondent illustrated:

*It is like training a child to talk*,* he/she will also repeat what you say*,* and so it is the same as this training; you have opened up our minds through training*,* especially some of us who never went to school.*

Many participants highly praised the Family MUAC program, and expressed their hope that it would continue for as long as possible. Finally, multiple participants described the difficulty in consistently taking MUAC measurements for their children alongside work responsibilities. Poverty emerged as a significant barrier and concern for families to secure the enduring well-being of their children’s health.

## Discussion

Our study investigated healthcare-seeking behaviors among caregivers of wasted children in two states in South Sudan, revealing higher care seeking and CMAM enrollment in Warrap compared to Central Equatoria, with an overall enrollment rate of 51.9%. Further analysis is needed to understand how contextual differences, such as urban versus rural settings, act as barriers or facilitators to care seeking. Although severe hunger was considerably higher in Warrap than in Central Equatoria (24.0% and 5.4%, respectively), the percentage of children ever wasted during the study period was higher in Central Equatoria than in Warrap (5.4% and 3.9% respectively). Despite Warrap experiencing higher hunger, flooding, and conflict, we hypothesize that children living in a more urban context (like Central Equatoria) can be exposed to unique risk factors that may not be as present in a more rural context (like Warrap).

While urbanization has increased employment opportunities for women living in Central Equatoria, women shoulder greater childcare responsibilities and burdens than their male counterparts [19]. To alleviate these burdens, caregivers often task older siblings with caring for their younger siblings, which may result in poorer childcare practices and is a risk factor for harm and injury [20]. Although more households in Warrap received food assistance, which may have offered some protection against wasting, targeted interventions tailored to urban and rural contexts could further improve outcomes, such as training older siblings to take MUAC measurements.

The primary barriers to care seeking, regardless of wasting status, included distance to the treatment centers, transportation, and treatment costs, and the findings are consistent with previous literature. A recent spatial accessibility analysis found that less than 30% of the population in Warrap State resided within five kilometers of a health facility, and less than 25% resided within a one-hour walk from a primary health care center [21]. Cost confounds the problem; the average cost of a one-way trip in South Sudan using local transportation is 1.50 USD [22], yet nearly 70% of the population lives on less than 2.15 USD per day [23]. Out-of-pocket spending for health and nutrition services is over 50% of the country’s total health sector expenditures; national and international government organizations fund a considerable percentage of the health sector [24]. Interventions that address some of the upstream and structural determinants of childcare practices could improve the uptake of desired practices. Community-based savings groups have been used effectively to help women overcome financial barriers in health care seeking in rural and fragile contexts, especially in emergencies [25, 26]. Integrating or co-locating Family MUAC programs with savings group initiatives could help mitigate treatment and transportation costs.

Caregiver knowledge and perceptions of health facilities also influenced care seeking, with 17.5% of caregivers in Central Equatoria citing a lack of awareness about where to seek care (no caregivers in Warrap reported this as a reason). While only 2.2% reported dissatisfaction with the quality of care, qualitative findings highlighted feelings of disrespect from health workers, potentially discouraging future visits. A recent study in South Sudan assessing maternal care health services found that women were afraid of facing dignity violations from health workers at a primary health care clinic and that these social fears may hold women back from seeking care [27]. Caregivers can be in a state of emotional vulnerability when seeking care for a malnourished or sick child, and studies have found a significant association between primary caregivers’ depression and their malnourished children in Botswana and Kenya [28, 29]. Training health workers in compassionate care could help reduce caregiver vulnerability and enhance care-seeking behavior.

Caregiver confidence in using MUAC tapes emerged as a key enabler of care seeking. Despite discouragement from others, most caregivers remained confident in their ability to take accurate measurements, with self-efficacy playing a motivational role in seeking care. In Ethiopia, caregivers with a high care-seeking ideation had an increased odds of prompt care seeking for children under five years with fevers [30]. We hypothesize the caregiver confidence in the MUAC tapes and their abilities to use the tapes correctly contributed to desired care-seeking practices.

In summary, our study provides novel insights by identifying the behavioral, social, and contextual factors that influence caregivers’ decisions to seek care after detecting child malnutrition using MUAC tapes—an aspect previously underexplored in the literature. It offers new evidence on the distinct challenges faced in urban and rural settings, such as competing childcare responsibilities in urban areas and financial constraints in rural areas, which influence care-seeking behaviors differently. Furthermore, the study underscores the critical role of caregiver empowerment and self-efficacy in sustaining MUAC measurement practices and highlights the need for tailored interventions that address both financial and social barriers to enhance program effectiveness.

### Strengths

The integration of the qualitative and quantitative findings provides a more comprehensive understanding of care-seeking behaviors by adding context to the observed trends. For example, while the quantitative analysis identified logistical barriers to care seeking such as transportation costs and distance to health facilities, qualitative insights revealed additional social and cultural factors, including caregivers’ lack of knowledge about available services and perceptions of care quality. Together, these findings offer a more nuanced perspective on the various factors influencing caregivers’ decisions to seek care.

### Limitations

This study is subject to at least three limitations. First, there is a possibility that social desirability and recall bias influenced caregivers’ reports of consistent use and high acceptability of MUAC tapes. To address these limitations, the study’s purpose was clearly explained during the consent process, and participants were encouraged to provide honest responses. Recall bias was further mitigated through triangulation by incorporating multiple sources, methods, and perspectives to enhance the credibility and validity of findings, utilizing open-ended and non-leading questions in the FGDs, and conducting discussions close to the time of the events being studied. Second, we could not ascertain actual care seeking, only reported care seeking, as records from CMAM sites could not be matched with caregiver responses. Finally, given that enrolled participants all lived within the catchment area of a CMAM program operated by a non-governmental organization, our findings may not be generalizable to other settings with more limited access to free malnutrition care services. The inherent limitations of qualitative research, which focuses on in-depth exploration of participants’ experiences and perspectives, further restrict the generalizability of our findings to broader populations or different contexts in South Sudan.

## Conclusion

Despite some caregivers reporting a lack of encouragement within their social networks, their high valuation and consistent use of MUAC tapes suggest a strong foundation for leveraging caregiver confidence to improve care-seeking behaviors. Future programs should build on this trust by developing targeted interventions that address key barriers such as contextual differences, financial constraints, and service quality. Integrating Family MUAC with community-based financial initiatives like savings groups can provide sustainable solutions to mitigate transportation and treatment costs, making care more accessible. Policymakers and practitioners should leverage formative research to tailor Family MUAC approaches to specific urban and rural contexts, ensuring that interventions effectively address the unique social and cultural norms that influence care-seeking behaviors. Investing in health worker training on compassionate and respectful care practices is essential to reducing caregiver hesitancy and fostering trust in health services, ultimately leading to improved child health outcomes. Finally, to ensure the long-term sustainability and impact of Family MUAC, future research should explore its influence on reducing child malnutrition rates over time and its potential to empower caregivers in making informed health decisions.

## Electronic supplementary material

Below is the link to the electronic supplementary material.

Supplementary Material 1

Supplementary Material 2

## Data Availability

The quantitative dataset supporting the conclusions of this study can be found in the Humanitarian Data Exchange at https://data.humdata.org/dataset/ssd-family-muac-mainpaper. Qualitative data is available upon request.

## Abbreviations

CDC: Centers for Disease Control and Prevention
CHW: Community health worker
CMAM: Community management of acute malnutrition
CNV: Community nutrition volunteer
FGDs: Focus group discussions
IRB: Institutional Review Board
LMICs: Low- and middle-income countries
MUAC: Mid-upper arm circumference
MOH: Ministry of Health
