# Supplementary material for: Exploring care-seeking practices within a family mid-upper arm circumference approach in South Sudan: a mixed-methods prospective study
Source: BMC Public Health. 2025 May 13;25:1751. doi: 10.1186/s12889-025-23010-w (PMC12070590; doi:10.1186/s12889-025-23010-w)
Supplement: Supplementary file 1 — Supplementary Material 1 [file 12889_2025_23010_MOESM1_ESM.docx]

**Annex A: South Sudan Focus Group Discussion Guide**

***Focus Group A – Organized by Community***

*Selecting focus group participants*: focus groups should ideally include 6 caregivers that have a range of experiences with: (1) households with malnourished children, (2) households that sought care, and (3) households on neither group #1 or group #2. Note: WV teams will be provided by CDC with a classification of participants according to frequency and presence of a malnourished child.

| **BACKGROUND INFORMATION** |  |
| --- | --- |
| FGD date |  |
| FGD facilitator name |  |
| Note taker name (if not audio recorded) |  |
| Location – State |  |
| Location – CMAM Site |  |
| Location – Boma/Community |  |
| MUAC TAPE GROUP |  Standard UNICEF  New UNICEF  GOAL |
| **CONSENT** | |
| Hello, my name is ____ and I represent World Vision and Johns Hopkins University. Your household previously agreed to participate in an evaluation of the World Vision Family MUAC program. The purpose of the evaluation is to understand if small changes, such as different measurement tapes, make it easier for families to monitor their child’s nutrition status. As part of the study, we are asking some caretakers that have been in the program to participate in a group interview. The purpose of this interview is to learn more about your experiences with the measurement tapes that were provided and experiences with care seeking for child malnutrition. By the end of the discussion, we hope to understand your experiences with the Family MUAC program and hear your recommendations for how the program could be improved.  The discussion today will be a group interview. We would like to gather as many different opinions and viewpoints as possible. If consensus is achieved on certain topics that is fine, but it is not necessary nor is it the goal. Rather the idea is to allow everyone to express their opinions and generate discussion among group members about these ideas. We expect that the discussion will last between 60 - 90 minutes.  The opinions you share in this discussion will remain confidential, and your name will not be recorded or linked to your responses. To maintain confidentiality, please do not discuss what group participants said with other people. There is a risk that some of the information you share could be repeated to others by another group member. If you agree, the interview may be audio recorded so that we can have a detailed record of the discussion for analysis; however, the information and perspectives you share in the interview will remain confidential and your name will not be recorded or linked to your responses.  You can decline to respond to a question or end the discussion at any time with no consequence. The information we collect will help us understand how to best deliver humanitarian assistance, but there are no direct benefits to participating. Your decision to participate and your responses will not negatively affect your participation in the Family MUAC program. Do you have any questions?  If questions come up later, you can always contact me or research coordinator (Daniel Atem Deng in Central Equatoria and Francis Obali in Upper Nile) at any time at this numbers (Central Equatoria: Daniel Atem Deng +211922088848; Warrap: Francis Obali +211926073929)  Do you agree to continue participating in the study? | |

| **PARTICIPANT INFORMATION** | | | | | | |
| --- | --- | --- | --- | --- | --- | --- |
| **Village of Residence** | **Participant** | | **Household Information** | |  |  |
|  | **Age** | **Sex** | **# of children <5yrs** | **Household Head Sex** | **Consent to participate** | **Consent to record** |
|  |  |  |  |  | Yes  No  | Yes  No  |
|  |  |  |  |  | Yes  No  | Yes  No  |
|  |  |  |  |  | Yes  No  | Yes  No  |
|  |  |  |  |  | Yes  No  | Yes  No  |
|  |  |  |  |  | Yes  No  | Yes  No  |
|  |  |  |  |  | Yes  No  | Yes  No  |
|  |  |  |  |  | Yes  No  | Yes  No  |
|  |  |  |  |  | Yes  No  | Yes  No  |
|  |  |  |  |  | Yes  No  | Yes  No  |

**1. Your experience with MUAC Tape**

1.1 We are interested in your experiences using the tape provided by WV for this project. Can you tell us how you like it, what about the tape you liked best? Is there anything you did not like?

- **Probe:** was it easy to use?
- **Probe:** Did you have any difficulty?
- **Probe:** Did it ever break or did it get lost?
- **Probe:** Was it well accepted by your child?

1.2 How could the MUAC tape provided by WV be improved?

- **Probe:** (**TAPE** **COLORS**) How do you feel about the colors of MUAC tapes with red for severe wasting, yellow for moderate wasting, and green for well-nourished? If you could design a MUAC tape, what colors would you use? Do you think another color (or colors) would be more helpful in understanding the results of the MUAC measurement?
- **Probe:** **(TAPE NUMBERS)** How do you feel about the size of the numbers? Are the numbers easy for you to understand?
- **Probe:** **(TAPE MATERIALS)** What materials would you use? Are the slits in the tapes easy to use? Why or Why not? Thickness of the tape? Durability of the tape?
- **Probe:** **(TAPE INSTRUCTIONS)** New UNICEF tape-specific probe**:** How often do you refer to the instructions on the back of the tape? Are the line drawings easy to follow? How often do you refer to them? Have you ever used them to teach another caregiver how to take a MUAC? How could the instructions be better and more easily understood?

1.3a ***Note to interviewer:***

*Have a table ready on a chart pre-filled with the attributes listed below. If caregivers provide additional attributes, add them to the chart. Once the group is comfortable that all key attributes are listed, go through a ranking exercise. For each attribute, all caregivers should mark how the tape they used performed on a scale from 5 (excellent) to 1 (very poor). Encourage discussion of different rankings.*

| Attribute | Caregiver 1 | Caregiver 2 | Caregiver 3 | Caregiver 4 | Caregiver 5 | Caregiver 6 |
| --- | --- | --- | --- | --- | --- | --- |
| *Durability of the material / doesn’t break or tear easily* |  |  |  |  |  |  |
| *Ease of keeping the tape safe from breaking, bending, or being damaged* |  |  |  |  |  |  |
| *Ease of keeping clean* |  |  |  |  |  |  |
| *Ease of use* |  |  |  |  |  |  |
| *Ease of using the slits* |  |  |  |  |  |  |
| *Ease of reading numbers (numeracy)* |  |  |  |  |  |  |
| *Ease of interpreting the meaning of the number* |  |  |  |  |  |  |
| *Ease of recognizing the colors* |  |  |  |  |  |  |
| *Ease of interpreting the meaning of the color* |  |  |  |  |  |  |
| *(ADD)* |  |  |  |  |  |  |

1.3b In thinking about what makes a MUAC tape better or worse, what attributes or characteristics do you think are important?

**2. Barriers to Regular Measurement**

2.1 We would like to know more about your MUAC measurement practice: how frequently do you measure your child’s MUAC? Have you established a routine around measuring MUAC? Can you describe it (e.g., when/ who/ where)?

- **Probe:** What motivates you to measure MUAC?
- **Probe:** Are there people who **encourage** you to take MUAC measurements? (e.g., your husband? Your mother or mother-in-law? Religious leaders? Health workers? Other caregivers? Friends?)
- **Probe:** Are there people who **discourage** you from taking MUAC measurements?

2.2 From the data we have collected, we saw that certain caregivers measure their children less often than weekly. Why do you think caregivers do not measure their children more often?

- **Probe:** Which barriers do you see as most common / experienced by most people?
- **Probe:** Have you personally experienced any of these barriers?
- **Probe:** With your current level of knowledge and skills, do you feel confident in using the MUAC tapes correctly to determine if your child is malnourished?

*Note to interviewer: We are interested to understand if there are barriers related to*

*(1)* ***Knowledge,*** *i.e., knowing what malnutrition is and why measuring MUAC is important,*

*(2)* ***Skills:*** *How to take the measurement, i.e., whether they feel* ***confident*** *using the MUAC tape and think they know how to make a measurement, and*

*(3)* ***Usefulness of the measurement,*** *i.e., whether they think there is value in measuring MUAC (e.g., if they identify the child as malnourished, they can access care).*

*If one of these three barriers isn’t brought up by respondents, prompt this barrier and see whether caregivers experience it.*

2.3 Do you think caregivers consider the measurements they take as accurate? Do caregivers trust their own results? Why or why not?

- **Probe:** Are they confident about the reading they make? Were the numbers familiar or easy to read? Were the colors easy to identify?
- **Probe**: Was the training adequate? Or have they received enough practice?

**3. Barriers to Care Seeking**

3.1 If you identified your child as malnourished (i.e., as having a yellow or red MUAC measurement), what would you do? Can you describe what actions – if any – you would take?

- **Probe:** How would you react to a yellow measurement? Would you seek care? Why or why not would you seek care?
- **Probe:** How would you react to a red measurement? Would you seek care? Why or why not would you seek care?
- **Probe:** Have you brought your children to the nutrition treatment center in the last 6 months? If you went, can you describe the experience?

3.2 Since receiving the MUAC tape and training from World Vision, has anyone that you know took their child to a nutrition treatment center been turned away? For example, have you heard of families that were told their child’s nutrition status was okay after they measured and thought they might be too thin? Please describe.

**4. Final remarks**

4.1. Do you have any advice for changes that we could make that would improve the Family MUAC program?

4.2 This is the end of the discussion. Would you like to share with us any additional thoughts?

**Phase I Focus Group Discussion – Comparison of Measurement Devices**

***Focus Group B – Organized with Members of All Communities***

*Selecting focus group participants*: focus groups should ideally include 6 caregivers including 2 caregivers assigned to use each of the 3 MUAC tapes evaluated as part of the study.

| **BACKGROUND INFORMATION** |  |
| --- | --- |
| FGD date |  |
| FGD facilitator name |  |
| Note taker name (if not audio recorded) |  |
| Location – State |  |
| Location – CMAM Site |  |
| Location – Boma/Community |  |
| **CONSENT** | |
| Hello, my name is ____ and I represent World Vision and Johns Hopkins University. Your household previously agreed to participate in an evaluation of the World Vision Family MUAC program. The purpose of the evaluation is to understand if small changes, such as different measurement tapes, make it easier for families to monitor their child’s nutrition status. As part of the study, we are asking some caretakers that have been in the program to participate in a group interview. The purpose of this interview is to learn more about your experiences with the measurement tapes that were provided and experiences with care seeking for child malnutrition. By the end of the discussion, we hope to understand your experiences with the Family MUAC program and hear your recommendations for how the program could be improved.  The discussion today will be a group interview. We would like to gather as many different opinions and viewpoints as possible. If consensus is achieved on certain topics that is fine, but it is not necessary nor is it the goal. Rather the idea is to allow everyone to express their opinions and generate discussion among group members about these ideas. We expect that the discussion will last between 60 - 90 minutes.  The opinions you share in this discussion will remain confidential, and your name will not be recorded or linked to your responses. To maintain confidentiality, please do not discuss what group participants said with other people. There is a risk that some of the information you share could be repeated to others by another group member. If you agree, the interview may be audio recorded so that we can have a detailed record of the discussion for analysis; however, the information and perspectives you share in the interview will remain confidential and your name will not be recorded or linked to your responses.  You can decline to respond to a question or end the discussion at any time with no consequence. The information we collect will help us understand how to best deliver humanitarian assistance, but there are no direct benefits to participating. Your decision to participate and your responses will not negatively affect your participation in the Family MUAC program. Do you have any questions?  If questions come up later, you can always contact me or research coordinator (Daniel Atem Deng in Central Equatoria and Francis Obali in Upper Nile) at any time at this numbers (Central Equatoria: Daniel Atem Deng +211922088848; Warrap: Francis Obali +211926073929)  Do you agree to continue participating in the study? | |

| **PARTICIPANT INFORMATION** | | | | | | | |
| --- | --- | --- | --- | --- | --- | --- | --- |
| **Village of Residence** | **Type of Tape Received** | **Participant** | | **Household Information** | |  |  |
|  |  | **Age** | **Sex** | **# children <5yrs** | **Household Head Sex** | **Consent to participate** | **Consent to record** |
|  |  |  |  |  |  | Yes  No  | Yes  No  |
|  |  |  |  |  |  | Yes  No  | Yes  No  |
|  |  |  |  |  |  | Yes  No  | Yes  No  |
|  |  |  |  |  |  | Yes  No  | Yes  No  |
|  |  |  |  |  |  | Yes  No  | Yes  No  |
|  |  |  |  |  |  | Yes  No  | Yes  No  |
|  |  |  |  |  |  | Yes  No  | Yes  No  |
|  |  |  |  |  |  | Yes  No  | Yes  No  |
|  |  |  |  |  |  | Yes  No  | Yes  No  |

***Instructions to Enumerators****: Start by providing all three tapes and allowing caregivers a chance to play with the tapes and practice taking measurements before starting the key informant interview. Allow at least 5 minutes for caregivers to use the tapes. Have a large chart or white board that can be used for the following ranking exercise.*

1.1 Can you describe your experience using the MUAC tape you received by WV?

- **Probe:** Which of these three tapes were you assigned for the study?
- **Probe:** Have you used any of the other tapes before?
- **Probe:** When were you first trained? How many times have you been trained?
- **Probe:** How often have you used the tapes?

1.2 ***Instructions to Enumerators****: help FGD participants identify characteristic and add them to the chart mentioned above which should be pre-filled with the attributes listed here. If caregivers provide additional attributes, add them to the chart. Once the group is comfortable that all key attributes are listed, go through a ranking exercise. For each attribute, all caregivers should mark how the tape they used performed on a scale from 5 (excellent) to 1 (very poor). Each caregiver should independently rank the tape they used. Encourage discussion of different rankings.*

| **Attribute** | **Caregiver 1** | **Caregiver 2** | **Caregiver 3** | **Caregiver 4** | **Caregiver 5** | **Caregiver 6** |
| --- | --- | --- | --- | --- | --- | --- |
| *Durability of the material / doesn’t break or tear easily* |  |  |  |  |  |  |
| *Ease of keeping the tape safe from breaking, bending, or being damaged* |  |  |  |  |  |  |
| *Ease of keeping clean* |  |  |  |  |  |  |
| *Ease of use* |  |  |  |  |  |  |
| *Ease of using the slits* |  |  |  |  |  |  |
| *Ease of reading numbers (numeracy)* |  |  |  |  |  |  |
| *Ease of interpreting the* ***meaning*** *of the number* |  |  |  |  |  |  |
| *Ease of recognizing the colors* |  |  |  |  |  |  |
| *Ease of interpreting the* ***meaning*** *of the color* |  |  |  |  |  |  |
| *(ADD)* |  |  |  |  |  |  |

**1.2a.** In thinking about what makes a MUAC tape better or worse, what attributes or characteristics do you think are important?

***Instructions to Enumerators****: After caregivers have individually evaluated each tape based on their own experience, encourage a discussion comparing the tapes based on prior experiences using the tapes as well as the use of the tapes on the day of the focus group. Put an X in the column for the tape that caregivers see as best for each attribute. If caregivers think that multiple tapes are similar with respect to the attribute, or if there is disagreement about which tape performs best, indicate that by putting an X in multiple columns and including some notes about the discussion.*

*If there are differences in the ranking (for example, one of the tapes is ranked highest in all attributes, while another received lower scores), ask the participants to elaborate further and find out what the understanding of the group is. Discuss whether the group tends to prefer one tape or another.*

| **Attribute** | **Traditional UNICEF Tape** | **New UNICF Tape** | **GOAL Tape** |
| --- | --- | --- | --- |
| *Durability of the material / doesn’t break or tear easily* |  |  |  |
| *Ease of keeping the tape safe from breaking, bending, or being damaged* |  |  |  |
| *Ease of keeping clean* |  |  |  |
| *Ease of use* |  |  |  |
| *Ease of using the slits* |  |  |  |
| *Ease of reading numbers (numeracy)* |  |  |  |
| *Ease of interpreting the* ***meaning*** *of the number* |  |  |  |
| *Ease of recognizing the colors* |  |  |  |
| *Ease of interpreting the* ***meaning*** *of the color* |  |  |  |
| *Durability of the material / doesn’t break or tear easily* |  |  |  |
| *(ADD)* |  |  |  |

1.3 We would like to talk with you about storage of the tapes: are there concerns with losing the tapes or having the tapes stolen?

- **Probe:** How do you store the tapes?
- **Probe:** What is the greatest threat to keeping the tapes safe (e.g., children playing with them? People stealing the tapes? Caregivers misplacing the tapes?)
- **Probe:** With respect to keeping the tape safe from loss or theft, is there a difference between the tapes?

1.4 How could the devices be improved?

- **Probe:** (**TAPE** **COLORS**) How do you feel about the colors of MUAC tapes with red for severe wasting, yellow for moderate wasting, and green for well-nourished? If you could design a MUAC tape, what colors would you use? Do you think another color (or colors) would be more helpful in understanding the results of the MUAC measurement?
- **Probe:** **(TAPE NUMBERS)** How do you feel about the size of the numbers? Are the numbers easy for you to understand?
- **Probe:** **(TAPE MATERIALS)** What materials would you use? Are the slits in the tapes easy to use? Why or Why not? Thickness of the tape? Durability of the tape?
- **Probe:** **(TAPE INSTRUCTIONS)** New UNICEF tape-specific probe**:** How often do you refer to the instructions on the back of the tape? Are the line drawings easy to follow? How often do you refer to them? Have you ever used them to teach another caregiver how to take a MUAC? How could the instructions be better and more easily understood?

1.5 Do you have any advice for changes that we could make that would improve the Family MUAC program?

1.6 This is the end of the discussion. Would you like to share with us any additional thoughts?
