# Supplementary material for: Exploring care-seeking practices within a family mid-upper arm circumference approach in South Sudan: a mixed-methods prospective study
Source: BMC Public Health. 2025 May 13;25:1751. doi: 10.1186/s12889-025-23010-w (PMC12070590; doi:10.1186/s12889-025-23010-w)
Supplement: Supplementary file 2 — Supplementary Material 2 [file 12889_2025_23010_MOESM2_ESM.docx]

**Annex B: South Sudan Family MUAC Caregiver Survey Questionnaire**

**Questionnaire Content – Baseline/Midterm/Endline & Monitoring Visits**

| **SECTION 1: BACKGROUND INFORMATION – ALL INTERVIEWS** | |
| --- | --- |
| 1.1 Interview date |  |
| 1.2 Enumerator name |  |
| 1.3 State | - Upper Nile - Central Equatoria |
| 1.4 CMAM Site | [list of names to be added] |
| 1.5 Boma | [list of names to be added] |
| 1.6 Village | [list of names to be added] |
| 1.7 Survey Type | - Enrollment - Midterm - Endline - Monitoring Visit |

| **SECTION 2: RESPONDENT INFORMATION AND CONSENT – ENROLLMENT INTERVIEW ONLY** | |
| --- | --- |
| *Recruit the primary caregiver of children <5 years of age, usually the mother (or if enrollment is at a family MUAC training event, the adult that presents with the child for training).* | |
| 2.1 Household ID number | Autogenerated/assigned at baseline |
| 2.2 Respondent name |  |
| 2.3 [TO BE READ ALOUD] Insert approved ENROLLMENT consent statement. | |
| 2.4 If you do not have further questions, may we proceed with the interview? | - Yes - No |
| 2.5 Location where interview is being conducted | - Family MUAC training site - Other community location (specify): - Respondent’s home |
| 2.6 Record interview location  *[Wait for few minutes. Accuracy distance of up to 5 meters is acceptable]* | [Coordinates] |

| **SECTION 3: RESPONDENT INFORMATION AND CONSENT – MIDTERM/ENDLINE/MONITORING INTERVIEW** | |
| --- | --- |
| 3.1 Household ID number | Record from list |
| 3.2 Respondent name | Record from list |
| 3.3 Does the name provided match the participant name from the list provided? | - Yes --> skip to consent statement - No |
| 3.4 Record which of the following situations best matches the situation | - Respondent is a family member of the original respondent. The original respondent died 🡪 skip to 3.5 - Respondent is a family member of the original respondent. The original respondent left the house for an extended time 🡪skip to 3.5 - Respondent is not a family member of the original respondent 🡪 End survey |
| 3.5 (Name) agreed to participate in an evaluation of World Vision’s Family MUAC program. We would like to ask a few questions about this today. Would you or someone else in your household consider participating on (name’s) behalf? If you say yes, we will record your name, explain about today’s interview and then you can decide about participating. | - Yes - No --> end interview |
| 3.6 Record replacement respondents name |  |
| 3.7 [TO BE READ ALOUD] Insert approved ABBREVIATED consent statement. | |
| 3.8 If you do not have further questions, may we proceed with the interview? | - Yes - No --> end interview |

| **SECTION 4: HOUSEHOLD INFORMATION – ENROLLMENT INTERVIEW ONLY** | |
| --- | --- |
| *We would like to begin the interview by asking some questions about your household.* | |
| 4.1 How many people live in this compound or house, and share food? Include infants, children, relatives and others that live here regularly. Do not include visitors that stay for one month or less. *(Enter 99 for don’t know/refused)* |  |
| 4.2 How many people in this compound/house are children less than five years of age?  *Time reference:* before month, year; show local calendar if needed to confirm number of children in age range. |  |
| 4.3 For how many of these children are you the primary caregiver?  *A primary caregiver is someone that prepares most of their food and shares a sleeping space, for example because the child has been orphaned.* |  |
| 4.4 Is the head of the household male or female? | - Male - Female |
| 4.5 Do you know the age of the household head? | - Yes - No --> skip to 4.7 |
| 4.6 How many years old is the household head? | ___ age in years (skip to 4.8)  ____ “don’t know” |
| 4.7 Please estimate the age of the household head using the time periods I will read aloud. | - Born before the 1^st^ civil war began (1962) – 59+ yrs - Born after the start of the 1^st^ civil war and before the 1^st^ peace agreement (1972) – 49-58 yrs - Born after the 1^st^ peace agreement and before the 2^nd^ civil war began (1983) – 38-48 yrs - Born after the 2^nd^ civil war began and before Sudanese military seized power (1989) – 32- 37 - Born after Sudanese military seized power and before the 2^nd^ peace agreement (2002) – 19-31 years - Born after the 2^nd^ peace agreement - ≤18 years - Don’t know |
| 4.9 What is the highest level of school the household head completed? | - No schooling - Attended but did not complete primary - Primary completed - Secondary completed - Don’t know |
| 4.10 How long has the household lived in this community? | - Less than 1 year - 1-5 years (more than one year ago but after Riek Machar was reinstated as vice president (April 2016) - 5-10 years (before Riek Machar was reinstated as vice president but after independence (2011) - More than 10 years (before independence) - Don’t know |
| 4.11 Is the household currently displaced, or have you ever been displaced?  *For example, forced to move due to conflict as internally displaced person (IDP) or refugee.* | - Never displaced - Returnee (formerly displaced from this area) - Currently displaced (conflict) from somewhere else - Currently displaced (non-conflict such as flooding, natural disaster) from somewhere else |

| **SECTION 5: PRIMARY CAREGIVER INFORMATION – ENROLLMENT INTERVIEW ONLY** | | |
| --- | --- | --- |
| *Now I would like to ask some questions about the primary caregiver of young children in the household. The primary caregiver is someone that prepares most of the food and shares a sleeping space with the child. The primary caregiver is usually the mother, but for example if a child has been orphaned it could be the father, another wife of the father, a grandmother or an older sibling.* | | |
| 5.1 Are you the primary caregiver for any children <5 years of age in the household? | | - Yes  - No |
|  | |  |
| 5.2 What is your relationship to the children <5 years of age in the household? *Check all that apply.* | | - Mother (biological or adoptive)  - Father (biological or adoptive)  - Older sibling  - Grandmother  - Other relative  - Other non-relative |
| 5.3 Did you receive a measurement device and training on how to measure the child’s arm to determine if they are malnourished? | | - Yes  - No  - Don’t know |
| 5.4 Did any other household members participate in training on measuring children’s arm size to determine if they have malnutrition? *Check all that apply.* | | - None 🡪 skip to 5.6  - Mother (biological or adoptive)  - Father (biological or adoptive)  - Older sibling  - Grandmother  - Other relative  - Other non-relative  *Skip pattern for all responses:*  *If 5.1 = no 🡪 5.5, If 5.1 = yes 🡪 5.6* |
|  | |  |
| 5.1 Are you the primary caregiver for any children <5 years of age in the household? | | - Yes (skip 5.5, 5.6, 7,8  - No |
| 5.5 Did the primary caregiver for any children <5 years of age in the household participate in the training? | | - Yes  - No  - Don’t know |
| *INTERVIEWER: INTERVIEWER: The following series of questions is about the primary caregiver. In most cases you will speaking to the primary caregiver. For interviews where you are speaking to another household member, invite the primary care giver to join the interview if they are available. If the primary caregiver is not available, be sure the information reported in the next section is about the primary caregiver (and not the respondent).* | | |
| Is the head of household the primary caregiver? | | - If yes, skip 5.11 |
| 5.6 Do you know your [primary caregiver’s] age? | | - Yes - No --> skip to 5.8 |
| 5.7 How many years old are you [is the primary care giver? | |  |
| 5.8 Please estimate your [the primary caregiver’s] age using the time periods I will read aloud. | | - Born before the 1^st^ civil war began (1962) – 59+ yrs - Born after the start of the 1^st^ civil war and before the 1^st^ peace agreement (1972) – 49-58 yrs - Born after the 1^st^ peace agreement and before the 2^nd^ civil war began (1983) – 38-48 yrs - Born after the 2^nd^ civil war began and before Sudanese military seized power (1989) – 32- 37 - Born after Sudanese military seized power and before the 2^nd^ peace agreement (2002) – 19-31 years - Born after the 2^nd^ peace agreement - ≤18 years - Don’t know |
| 5.9 Have you [the primary caregiver] ever attended school? | | - Yes - No --> skip to 5.11 - Don’t know --> skip to 5.11 |
| 5.10 What is the highest level of schooling completed? | | - Attended but did not complete primary - Primary completed - Secondary completed - Don’t know |
| 5.11 What is your [the primary caregiver’s] current marital status? | | - Married / in a long-term relationship  - Divorced / separated  - Widowed  - Not married / single   - Refused / No answer |
| 5.12 Is the primary caregiver male or female?  *If respondent is primary caregiver, do not ask aloud.* | | - Female - Male --> skip to next section |
| 5.13 Are you currently pregnant? | | - Yes - No - Unknown |
| 5.14 Are you currently breastfeeding? | | - Yes - No |
| **SECTION 6: HOUSEHOLD ECONOMY AND RECEIPT OF HUMANITARIAN ASSISTANCE –**  **ENROLLMENT/MIDTERM/ENDLINE INTERVIEWS** | | |
| *Now I would like to ask some questions about your households spending and income sources.* | | |
| **6.1 HOUSEHOLD EXPENDITURES**. Please estimate your household’s expenses (in SSP) the PAST MONTH for each of the following categories. Enter 99 if unknown for module as a whole. | | |
| A. Housing | |  |
| B. Fuel (for example, charcoal for cooking, gas for generator) | |  |
| E. Food | |  |
| C. Household items | |  |
| D. Transportation | |  |
| E. Health care / medicine | |  |
| F. Education | |  |
| G. Debt | |  |
| H. Other | |  |
| 1. Were your household's expenditures in the past month similar to your usual expenditure levels? | | - Yes - No, lower than usual - No, higher than usual - Don't know - Refused / no answer |
| **6.2 HOUSEHOLD INCOME**. Now I would like to ask some questions about your household’s income in the past month. | | |
| 1. What is the household’s main sources of income? | - Crop farming - Animal husbandry - Owned business (e.g. shop, factory) - Wages and salaries - Remittances - Humanitarian assistance - Other | |
| 1. For the last month, what was the total household income (in SSP)? *Enter 999 for don’t know/refused.* | ___ SSP  “don’t know” (skip to D) | |
| 1. Was your household's income in the past month similar to your usual income level? | - Yes - No, lower than usual (explain) - No, higher than usual (explain) - Don't know / Refused | |
| 1. How much does your household currently have in savings (in SSP)? *Enter 99 for don’t know/refused.* |  | |
| 1. How much debt does your household currently have? (in SSP)? *Enter 99 for don’t know/refused.* |  | |
| **6.3 HUMANITARIAN ASSISTANCE.** *Now I would like to ask some questions about if your household has recently received any humanitarian assistance, and the types of assistance recently received.* | | |
| 1. Within the PAST 3 MONTHS, has your household received any type of humanitarian assistance that was either food, a food voucher or a cash transfer? | - Yes - No --> skip to next section | |
| 1. Do you receive assistance regularly, for example the same amount each month? | - Yes - No | |
| 1. What type(s) of assistance did your household receive in the past 3 months? *Multiple responses permitted.* | - In kind food aid (if no, skip E and F) - Water, sanitation, and/or hygiene - Livelihoods - Food voucher - Currency / cash (if no, skip D) - Other (e.g., mobile phone transfer, e-voucher) - Don't know / Refused | |
| 1. What is the total amount of cash or vouchers received in the most recent month of assistance?   *Record response in SSP; enter 99 for don’t know/refused.* |  | |
| 1. Please estimate total amount of in-kind food assistance (e.g. GFD) received in the most recent month of assistance? *Record response in kg; enter 99 for don’t know/refused.* |  | |
| 1. In the most recent month of assistance, do any children in your household receive extra food or special foods (e.g. BSFP)?  *Question relates to products specifically for children 6-59 months.* *Show examples of common items.* *Check all that apply.* | - Extra food for children - Liquid nutrition supplements for children - High energy biscuits for children - Other (specify):____________________________ | |

| **SECTION 7: FOOD SECURITY** | |
| --- | --- |
| 7.1 How many meals did your household consume YESTERDAY? |  |
| **7.3 HOUSEHOLD HUNGER SCALE** | |
| A. In the past MONTH, was there ever no food or not enough food to eat (of any kind) in your household because of lack of resources? | - Yes - No --> skip to 6.3b - Don’t Know --> skip to 6.3b |
| B. How often did this happen in the past month? | - Rarely (1-2 times) - Sometimes (3-10 times) - Often (more than 10 times) - Don’t know/remember - Refused/no answer |
| C. In the past MONTH, did you or any household member go to sleep at night hungry because there was not enough food? | - Yes - No --> skip to 6.3d - Don’t Know --> skip to 6.3d |
| D. How often did this happen in the past 30 days or since this time last month? | - Rarely (1-2 times) - Sometimes (3-10 times) - Often (more than 10 times) - Don’t know/remember - Refused/no answer |
| E. In the past MONTH, did you or any household member go a whole day and night without eating anything at all because there was not enough food? | - Yes - No --> skip to next section - Don’t Know --> skip to next section |
| F. How often did this happen in the past 30 days or since this time last month? | - Rarely (1-2 times) - Sometimes (3-10 times) - Often (more than 10 times) - Don’t know/remember - Refused/no answer |

| **SECTION 8: CHILD SUB-FORM (complete one per child) – ENROLLMENT INTERVIEW ONLY AND AT MIDTERM FOR CHILDREN IN THE HOUSEHOLD NOT PREVIOUSLY ENROLLED THAT MEET STUDY CRITERIA.** | | |
| --- | --- | --- |
| *We would like to ask you some questions about your children. For each child less than five years of age, we will ask several questions and also take a measurement of their arm. Please make sure that all children that you care for are included, even if they are not biologically related.*  *At midterm: complete this form for any new children that are eligible, either because they moved into the household or because they are now between 5-53 months of age.* | |  |
| 8.1 What is his/her name? |  |  |
| 8.2 What is the child’s sex? | - Male - Female |  |
| 8.3 Does this child have a health card, and if so, may I see it? | - Yes, health card presented - Yes, has health card but not available at interview - Child does not have health card - Refused to share health card/respond - Don’t know |  |
| 8.4 How old is [name]?  *Report age in months, enter 99 for don’t know* | *­­­­­­­*  *Values should be <54 months* | |
| 8.5 Do you know his/her month and year of birth?  *[Ask to see a vaccination / health card]* | - Yes, parent reported (unconfirmed) - Yes, confirmed with health/vaccination card - No, birth date unknown -->8.7 | |
| 8.6 Indicate the date of birth | *Day_______ Month _______ Year _______*  ***do not require day*** | |
| 8.7 How many siblings does this child have?  *Note: siblings include all children in the household (biological or not)* | - *0* - *1* - *2* - *3* - *4* - *5* - *6 or more* | |
| 8.8 Has the child ever been diagnosed or treated for acute malnutrition? (per caregiver report)  *For example, due to being too thin or not having enough food, did the child receive specialized foods and nutrition checks until they were recovered? This service could have been provided at a nearby health facility or by World Vision or another NGO.* | - Yes - No --> skip to 8.13 - Don’t know --> skip to 8.13 | |
| 8.9 What year were they most recently diagnosed or treated for acute malnutrition? | - 2022  - 2021  - 2020  - 2019  - 2018 or before  - Don’t know | |
| 8.10 Were they treated and did they fully recover? | - Diagnosed but did not receive any treatment  - Received treatment and recovered  - Received treatment but did not recover  - Don’t know | |
| 8.11 Was this the only time the child was diagnosed with acute malnutrition? | - Yes - No - Don’t know | |
| 8.12 How many times has the child been diagnosed with malnutrition before? *Enter 99 for don’t know.* |  | |
| *Now I would like to check the nutrition status of your child. First I will ask you to measure the child’s arm and then I will also take a measurement. If the child is malnourished, I will provide information on where you should take them for treatment.* | | |
| 8.13 **HOUSEHOLD MUAC MEASUREMENT** – using measurement tape provided by WVI Family MUAC program.  *Note: if at midterm, device is not available, allow measurement using the device brought by the data collector.* | | |
| 1. Device type   *(record to nearest 0.1 cm)* | - Standard UNICEF tape - New UNICEF tape - GOAL tape 🡪 skip to 8.13c | |
| 1. Arm circumference measurement   *(record to nearest 0.1 cm)* | *If child is not present, record 99*  *If care taker is illiterate, record 98*  *If measurement cannot be take for another reason, record 97* | |
| 1. Mid-upper arm measurement color | - Green - Yellow - Red - Child not present | |
| 8.14 **DATA COLLECTOR MEASUREMENT** – using device provided by WVI Family MUAC program. | | |
| 1. Mid-upper arm circumference   *(record to nearest 0.1 cm)* | *If child is not present, record 99* | |
| 1. Mid-upper arm measurement color | - Green - Yellow - Red - Child not present | |
| C. Bi-lateral pedal edema | - Yes - No - Child not present | |
| D. Referral status (per data collector measurement) | - No referral needed - Referred to WV CMAM site (nutrition evaluation)   *If yes, referral ticket number: ___________*   - Refer to health facility (other symptoms of illness) | |

| **SECTION 9: Family MUAC (MIDTERM/ENDLINE/MONITORING VISITS ONLY)** | |
| --- | --- |
| *We are interested in learning about your experience with nutrition programs and would like to finish our interview by asking a few more questions on this topic.* | |
| 9.1 Did your household receive a tape/bracelet that is for measuring your child’s arm?  *Interviewer: this should be the device provided by World Vision within the past year. Do not include other devices that may have been previously received.* | - Standard UNICEF tape  - New UNICEF tape  - GOAL tape  - No tape received  - Don’t know |
| 9.2 Can you please show me the tape? | - Tape available -->--> skip to 9.4  - Tape not available |
| 9.3 Why is the tape not available? | - Lost  - Broken or discarded because it was broken  - Discarded because it was not wanted/useful  - Given away  - Sold  - Interview conducted outside home / forgot to bring  - Other *specify* ________________________________ |
| 9.4 What type(s) of damage does the tape have?  *Check all that apply.* | - No damage - Bent, creased or permanent fold lines - Broken into one or more pieces or significantly torn - Closure area is damaged (e.g. slot or area of device that is inserted into the slot) - Colors faded - Cannot read writing   Other (specify) |
| 9.5 Is the device still functional?  *[Interviewer assessed, do not ask]* | - Yes, tape is functional --> skip to 9.5  - No, tape is broken or damaged |
| 9.6 How often do you or someone in your households use the device to measure the arm circumference of children in your household? | - At least every week --> skip to 9.10  - Several times a month (but not weekly) --> skip to 9.10  - About once a month -->--> skip to 9.10  - At least once in the past 2 months  - More than 2 months ago   - - Don’t know |
| 9.7 Why don’t you use the tape more often?  *Do not read responses aloud. Check all that apply.* | - It is too difficult to use  - I am worried I might measure incorrectly  - My child doesn’t like it  - I don’t have enough time  - I forget to use it regularly  - My spouse/family is not supportive  - No longer have the device (lost, stolen, sold)  - Device is broken or damaged  - Not enough training on how to use device  - Do not know what to do if child is malnourished  - Prefer to rely on CNV/CHW/others for screening  - Other *specify* ________________________________ |
| 9.8 Do you feel comfortable measuring arm circumference using this device? | - Yes 🡪 skip to 9.8  - No  - Don’t know |
| 9.9 Why are you uncomfortable or uncertain about using the tape? |  |
| 9.10 Within the past two months, has your household received any visits by a community health worker or nutrition volunteer? | - Yes, CNV visit(s)  - Yes, CHW visit(s)  - No --> skip to next section  - Don’t know --> skip to next section |
| 9.11 Did they check the child’s nutrition status? | - Yes  - No  - Don’t know |
| 9.12 Did they provide any training on how to measure your child’s arm or check their nutrition status? | - Yes  - No  - Don’t know |
| 9.13 Did they provide any advice on what to do if you think your child is malnourished? | - Yes  - No  - Don’t know |
| 9.14 If your MUAC measurement device was lost or broken, did they provide a replacement? | - Device was not lost/broken  - Yes, a replacement was provided  - No, a replacement was not provided (still needed)  - Don’t know |
| 9.15 Would you like these visits to continue? | - Yes--> skip to next section - No - Don’t know --> skip to next section |
| 9.16 Why do you not want to continue with visits? |  |

| **SECTION 10: CHILD SUB-FORM (complete one per child) – MIDTERM/ENDLINE/MONITORING VISITS** | |
| --- | --- |
| *We would like to ask you some questions about your children. For each child less than five years of age, we will ask several questions and also take a measurement of their arm. Please make sure that all children that you care for are included, even if they are not biologically related.* | |
| 10.1 What is his/her name? *Confirm from list.* |  |
| 10.2 What is [child]’s age in months?  *Confirm from list.* |  |
| 10.3 Child Unique ID. *Record from list.* |  |
| 10.4 Visit type | - Monitoring visit 🡪 skip to 10.6  - Midterm or endline visit |
| *Now I would like to check the nutrition status of your child. First I will ask you to measure the child’s arm and then I will also take a measurement. If the child is malnourished, I will provide information on where you should take them for treatment.* | |
| **10.5** **HOUSEHOLD MUAC MEASUREMENT** – using device type provided by WVI Family MUAC program.  *Now I would like to check the nutrition status of your child. First I will ask you to measure the child’s arm and then I will also take a measurement. If the child is malnourished, I will provide information on where you should take them for treatment*  *Note: if device is not available, allow measurement using the device brought by the data collector.* | |
| 1. Device type   *(record to nearest 0.1 cm)* | - Standard UNICEF tape - New UNICEF tape   GOAL tape 🡪 skip to 10.5C |
| 1. Arm circumference measurement   *(record to nearest 0.1 cm)* | *If child is not present, record 99*  *If care taker is illiterate, record 98*  *If measurement cannot be take for another reason, record 97* |
| 1. Mid-upper arm measurement color | - Green - Yellow - Red - Child not present |
| **10.6** **DATA COLLECTOR MUAC MEASUREMENT** – using device provided by WVI Family MUAC program. | |
| 1. Mid-upper arm circumference   *(record to nearest 0.1 cm)* | *If device does allow cm measurement, record 98*  *If child is not present, record 99* |
| 1. Mid-upper arm measurement color | - Green - Yellow - Red - Child not present |
| 1. Bi-lateral pedal edema | - Yes - No - Child not present |
| 1. Referral status   (per data collector measurement) | - No referral needed - Referred to WV CMAM site (nutrition evaluation)   *If yes, referral ticket number: ___________*   - Refer to health facility (other symptoms of illness) |
| **Questions for Caregivers** | |
| 10.7 In the past two months, did you ever measure [child]’s arm to see if they were too thin? | - Yes - No -->skip to 10.9 - Don’t know --> skip to 10.9 |
| 10.8 When you measured [child], did you ever determine they were too thin? | - Yes - No - Don’t know |
| 10.9 In the past two months, did you take [child] for care because you were worried about their nutrition, not eating enough or being too thin? | - Yes, health facility - Yes, nutrition program site - Yes, community health/nutrition volunteer - Yes, traditional healer--> skip to 10.15 - No --> skip to 10.15 - Don’t know |
| 10.10 During your consultation (or at the facility if referred by a CHW/CNV) did they measure [child]’s arm or weight? | - Yes - No --> skip to next section - Don’t know --> skip to next section |
| 10.11 Was [child] determined to be too thin or have malnutrition? | - Yes - No --> skip to next section - Don’t know --> skip to next section |
| 10.12 Is [child] currently receiving treatment for malnutrition? | - Yes - No --> skip to next section - Don’t know --> skip to next section |
| 10.13 Where is [child] receiving treatment? | - World Vision CMAM Site - Other nearby health facility/CMAM site - CHW, CNV or mobile team that visits community - Other |
| 10.14 Can you tell me more about where [child] is receiving treatment? (e.g. facility/organization name, location) | *Open text field.* |
| 10.15 Why did you not take [child] for care at health facility or nutrition treatment site?  *Do not read aloud. Record only one response – if multiple reasons mentioned, probe for most important.* | - Child was not malnourished - Did not know where to go - Did not like quality of care - Treatment center was far away - Could not reach treatment center (e.g. roads impassible, poor security) - Too expensive [transportation] - Too expensive [treatment cost] - Treatment center hours are not convenient - Did not have time / too busy - Family did not think it was important to go - Other: __________________________________ |

| This is the end of the questions. Thank you for your answers. Do you have any questions for me? |
| --- |
